# Interventions addressing maternal and child health among the urban poor and homeless: an overview of systematic reviews

**DOI:** 10.1186/s12889-023-15410-7

**Published:** 2023-03-14

**Authors:** Devaki Nambiar, Bincy Mathew, Shubhankar Dubey, Sandeep Moola

**Affiliations:** 1 The George Institute for Global Health, 308 Elegance Tower, Jasola District Centre, 110025 New Delhi, India; 2 Independent Researcher, New Delhi, India; 3 Indian Council of Medical Research- Regional Medical Research Center, Bhubaneswar, Odisha India; 4 Sanofi Healthcare India Pvt Ltd, Hyderabad, India

**Keywords:** Urban poor, Homeless, Maternal and child health, Low- and Middle-Income Countries, LMIC, Systematic review, Overview, Umbrella review

## Abstract

**Background:**

Inequalities in access to and utilization of maternal and child health (MCH) care are hampering progress on the path to achieving the Sustainable Development Goals. In a number of Low- and Middle-Income Countries (LMICs) population subgroups at disproportionate risk of being left behind are the urban poor. Within this neglected group is the further neglected group of the homeless. Concomitantly, a number of interventions from the antenatal period onward have been piloted, tested, and scaled in these contexts. We carried out an overview of systematic reviews (SRs) to characterize the evidence around maternal and child health interventions relevant to urban poor homeless populations in LMICs.

**Methods:**

We searched Medline, Cochrane Library, Health Systems Evidence and EBSCOhost databases for SRs published between January 2009 and 2020 (with an updated search through November 2021). Our population of interest was women or children from urban poor settings in LMICs; interventions and outcomes corresponded with the World Health Organization’s (WHO) guidance document. Each SR was assessed by two reviewers using established standard critical appraisal checklists. The overview was registered in PROSPERO (ID: CRD42021229107).

**Results:**

In a sample of 33 high quality SRs, we found no direct relevant evidence for pregnant and lactating homeless women (and children) in the reviewed literature. There was a lack of emphasis on evidence related to family planning, safe abortion care, and postpartum care of mothers. There was mixed quality evidence that the range of nutritional interventions had little, unclear or no effect on several child mortality and development outcomes. Interventions related to water, sanitation, and hygiene, ensuring acceptability of community health services and health promotion type programs could be regarded as beneficial, although location seemed to matter. Importantly, the risk of bias reporting in different reviews did not match, suggesting that greater attention to rigour in their conduct is needed.

**Conclusion:**

The generalizability of existing systematic reviews to our population of interest was poor. There is a clear need for rigorous primary research on MCH interventions among urban poor, and particularly homeless populations in LMICs, as it is as yet unclear whether the same, augmented, or altogether different interventions would be required.

**Supplementary Information:**

The online version contains supplementary material available at 10.1186/s12889-023-15410-7.

## Background

Inequality in access to maternal healthcare services has consequences for Sustainable Development Goals (SDG) target 3.1 and 3.2, which relate to reduction of the global maternal mortality ratio, neonatal mortality and under-5 mortality, respectively [1]. Globally, 42 countries in the Sahel (above the Sahara desert through to the West African coast), Sub Saharan, South Asian, South East-Asian and parts of South American regions are unlikely to attain SDG targets for maternal and child mortality [2].

By the last year of SDG, i.e. 2030, it is estimated that 60 per cent of people will live in cities [3]. Moreover, with the global expansion of towns and cities, there has for some time been a trend of urbanization of poverty [4]. The convenience of living in cities (shorter distances, accessibility of services, social networks) benefits a small percentage of population with millions of urban-dwellers being excluded [3]; which in turn has implications for maternal [5] and child health [6]. A study drawing from Demographic and Health Surveys in Least Developed Countries showed significant inequalities in children’s nutritional outcomes, with higher inequalities in the most rapidly urbanizing countries [7].

Despite inequalities in health outcomes among the urban poor, issues relating to access persist. A study of 22 African cities showed disruptions in the maternal continuum of care, characterized drop offs in antenatal care, childbirth and postpartum care, with varying reliance on public and private sector use, and use of hospitals across cities [8]. Another study, drawing on data from seven cities in LMICs highlighted issues relating to availability, accessibility, quality of MCH services as well as delayed care-seeking [5]. In a study on 30 developing countries, it was noted that the urban poor did not have better access to maternal healthcare despite proximity to healthcare services [9]. Among the urban poor, people living on the streets are at the highest risk of being left behind, because they are hard to reach and often are not covered by social welfare systems [10]. Thus, homeless people have disproportionately higher levels of morbidity and mortality compared to the general population, [11, 12] and the homelessness of pregnant women is associated with poor health outcomes of children [13, 14].

Interventions introduced from the antenatal period to the later childhood period can bring about a decrease in neonatal and later mortality [15]. Interventions relating to the health sector including those outside it are needed to bring countries on track for achieving SDG goals 3.1 and 3.2. These include scaling up of integrated packages of reproductive health, maternal and newborn health, and child health as well as those beyond it such as access to clean water and sanitation [2].

Maternal and child health interventions have been a major focus of public health research for decades and are in a sense core to the discipline itself. Drawing on this evidence base, a multitude of systematic reviews have assessed a broad range of interventions, with varying primary aims and outcomes recognize the potential of interventions to improve maternal and child health outcomes [16–18]. We sought to compile an overview of SRs to examine and consolidate evidence on the interventions relating to clinical, public health or community-based health or health promotion services in context of maternal and child health in LMICs, with focus on homeless populations.

## Methods

An overview of SRs was conducted based on a pre-defined protocol, which was registered in PROSPERO (ID: CRD42021229107, the full protocol may be downloaded at this link).

### Inclusion criteria

Systematic reviews involving women and children from urban poor settings in LMICs, including the homeless were included. Populations of interest were women (pregnant or lactating women, if any) or children from urban poor settings in LMICs. We were looking for any interventions (clinical or public health or community-based health or health promotion, etc.) addressing MCH were considered, guided by the WHO guidance document on packages of interventions for family planning, safe abortion care, maternal, newborn and child health [19]. Comparator interventions included usual or standard care, no intervention, or another intervention. Outcome indicators were also aligned with the WHO guidance document [19] pertaining to family planning, safe abortion care, pregnancy care, childbirth care, postpartum care of the mother, care of the newborn, and care during infancy and childhood were considered. Some of the outcome indicators included unmet need for family planning, percentage of health providers trained to provide safe abortion, percentage of pregnant women receiving antenatal care at least once/four times during pregnancy, percentage of births in facilities, percentage of women receiving postpartum care within seven days after childbirth, neonatal and early neonatal mortality rates, and percentage of infants under six months exclusively breastfed.

All the SRs with or without meta-analysis of any study design were included. Non-English language reviews were considered for inclusion where English translated reviews were available. However, the overview did not find any SRs published in non-English languages. All the available SRs published from January 2009 till January 2020 were considered. An updated search was conducted from February 2020 till November 2021, utilising the same search strategies. Reviews that incorporated theoretical studies or text or opinion as the primary source of evidence were excluded. Reviews that included interventions conducted only in high-income countries (HICs) and those that had interventions in general women and child populations (i.e., non-urban) conducted in LMICs were excluded.

### Search methods and review selection process

**Fig. 1:**
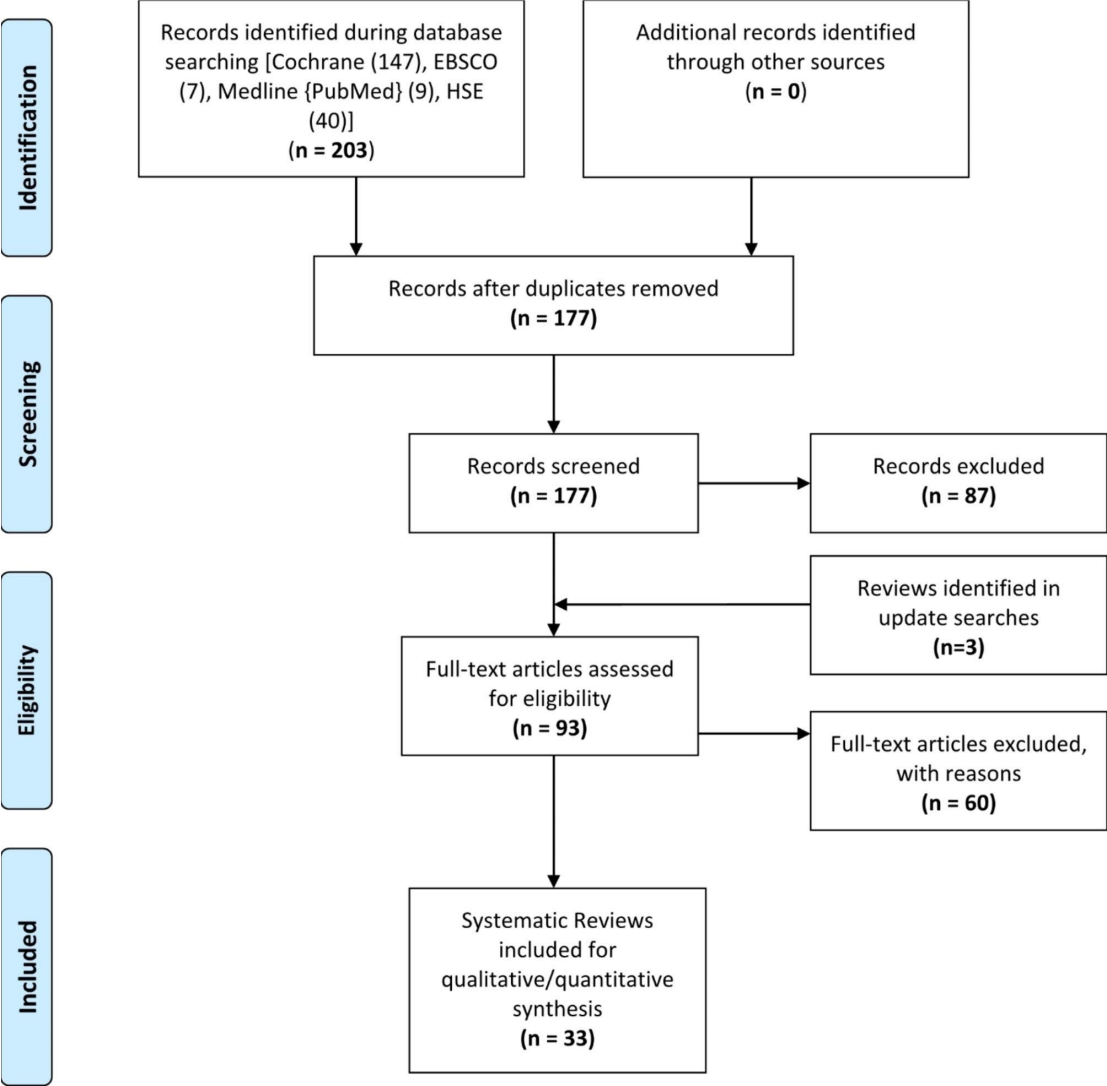
PRISMA Flow diagram

A comprehensive literature search was conducted in databases such as Medline (PubMed), Cochrane, Health System Evidence (HSE) and EBSCOhost platform. The search strategies are available in Supplementary File 1. Two reviewers (SD, BM) independently performed preliminary screening of titles and abstracts of the records with support from SM. Full-text screening of the selected records was done independently by SD and BM. Conflicts were resolved with mutual consensus and consultation with a third reviewer (DN). The review selection process is presented in the flow diagram adapted from the Preferred reporting items for systematic reviews and meta-analyses (PRISMA) guidelines (see Fig. 1) [20].

### Assessment of methodological quality of included reviews

The methodological quality of each included SR was assessed in duplicate by SD and BM using A Measurement Tool to Assess Systematic Reviews (AMSTAR-2) checklist [21] and Joanna Briggs Institute (JBI) critical appraisal checklist [22]. The quality rating in the included SRs was classified as high, moderate, low depending on the scores for individual items in the tools as decided with consensus (High: if Yes > 81%; Moderate: between 81% and 56%, Low: if Yes < 60%). Resolution of any disagreements was resolved through consensus with the help of a third reviewer (DN).

### Data extraction and synthesis

The data were independently extracted by SD and BM using a predesigned data extraction template. The following data were extracted: author/year, objectives, review characteristics, description of interventions and comparators, outcomes, and results. Extracted findings were summarised narratively by population and intervention type. Where SRs assessed certainty of evidence using the Grading of Recommendations, Assessment, Development and Evaluations (GRADE) [23] and GRADE-CERQual approaches, [24] for quantitative and qualitative reviews, respectively, we reported the findings accordingly. The GRADE approach is used in individual SRs to assess the quality of evidence or the confidence in the effect estimates [25].

## Results

Based on a comprehensive search performed, 203 reviews were identified. After removing duplicates, 177 records underwent title and abstract screening, of which 87 were excluded. Of the 93 records remaining, 54 records were excluded during the full-text screening of the articles. Thirty-three high-quality reviews were finally included for this review (see Fig. 1 for PRISMA flow diagram). This included three new SRs identified in the updated searches [26–28].

Most of the reviews included infants and children from urban poor settings. Other participants included pregnant and lactating women. The reviews addressed family planning, pregnancy care, postpartum care of the mother and care of the newborn, childbirth care, And care during infancy and childhood. None of the reviews addressed abortion care.

Based on the eligibility criteria, among the 39 reviews, two were of low quality, [29, 30] four were of moderate quality, [15, 31–33] and the remaining 33 were of high quality. We included only high-quality reviews (N = 33) as adjudged during critical appraisal. Of the 33 SRs, 29 used meta-analysis, mainly comprising randomized controlled trials (RCTs) and non-randomized and quasi-experimental designs. Three reviews did not conduct a meta-analysis, and one review was a qualitative synthesis. The three new SRs identified in the updated searches examined hand washing promotion, education of family members to support weaning, and targeted client communication via mobile devices [26–28].

### Summary of key findings

#### Interventions relevant to specific urban poor populations (i.e., the homeless, slum dwellers)

Overall, six reviews included studies relevant to urban slum settings; however, most of the evidence was based on single studies from the reviews; hence the findings should be interpreted with caution [34–39]. In the Turley et al. (2013) review, there was low quality, but consistent body of evidence to indicate that slum upgrading – defined as “improving the physical environment, for example the water supply, sanitation, waste collection, electricity, drainage, road paving and street lighting” (p.3) may reduce the incidence of diarrheal diseases [34]. Moderate quality evidence was found for that supplementary feeding improving the physical growth of middle income children as compared to slum-dwelling children in India; authors opine that the differential effects may have been due to poor environmental conditions for slum-dwelling children that in turn decreased the effectiveness of the intervention [35]. Moderate quality evidence showed that the Sanitation Hygiene Education and Water Supply in Bangladesh (SHEWA-B) programme resulted in little to no difference on diarrhea prevalence among children (aged < five years) living in urban slums [37].

Moderate quality evidence indicated beneficial effect of nutritional interventions on length at birth and low birth weight (LBW); however, low quality evidence indicated that the interventions may reduce stunting in infants and children below five years of age in locations outside slum areas [39]. The review by Lassi et al. (2016) found that there was low quality evidence that zinc supplementation may reduce the incidence and prevalence of pneumonia among children under five years in low-income urban areas in LMIC contexts, including slums in India [36].

A qualitative review of factors influencing antenatal care (ANC) visits found that pregnancy was seen as a healthy condition by women in a slum in Dhaka that rendered ANC visits unnecessary, and this finding was based on high confidence in the evidence [38].

#### Interventions relevant to urban poor populations in general

Among the included SRs, 14 reviews were on nutritional interventions, 15 on community health services (e.g., immunization) and health promotion, while three reviews were on water sanitation and hygiene (WASH) and one on slum upgrading. Findings from these reviews are summarized below.

#### Nutritional interventions

Most reviews reported on nutrient supplementation for improving MCH. Some interventions assessed supplementation (lipid-based nutrients, vitamin A, vitamin D), others looked at fortification, and the remaining examined supplementary feeding.

### Child growth

Moderate quality evidence indicated that lipid-based nutrient supplements (LNS) (given to pregnant women) might be of slight benefit to babies who are born small, as well as on newborn weight and length compared to iron-folic acid (IFA) [40]. In another review, low quality evidence indicated that multiple micronutrient (MMN) fortification may improve child growth, measured as a weight for age and height/length [41]. Low quality evidence showed that micronutrient or macronutrient supplementation of children (birth to 59 months) had little to no effect on height for age (HFA) and on length. There was no evidence of an effect of zinc supplementation given to pregnant women on LBW and length [39]. Low quality evidence from another review suggested that supplementary feeding had little to no effect on child growth in children under five years [42].

Very low quality evidence indicated that providing additional food to children aged three months to five years may lead to small gains in weight (0.24 kg a year) and height (0.54 cm a year) [35]. Specifically, this review indicated that “Supplementary feeding young children has a small effect on gain in weight and weight-for-age z-scores (WAZ) in low- and middle-income countries… Supplementary feeding for young children has a small effect on linear growth in low- and middle-income countries… Supplementary feeding may have a moderate positive effect on psychomotor development in low- and middle-income countries… The evidence of effects on cognitive development in low- and middle-income countries is sparse and mixed….” [35] Limited evidence suggested little to no effect of animal-source food compared to cereal products or no intervention on growth outcomes of children [43]. Another SR showed that compared to specially formulated fortified foods, LNS might be slightly more effective in aiding recovery from MAM and effective in weight gain among children aged 6 to 59 months [44]. Low to moderate quality evidence showed that nutrition education to families about appropriate feeding practices during weaning may slightly improve weight and height at 12 months of age [27].

### Anemia and vitamin deficiency

Moderate quality evidence suggested that IFA and MMN likely resulted in a decrease in maternal anemia compared to LNS [40]. Low quality evidence indicated that fortified rice with only iron or in conjunction with other micronutrients might make little to no difference to the risk of having anemia. However, the intervention might decrease the risk of iron deficiency in children, non-pregnant and non-lactating women [45]. Low quality evidence from another review indicated that MMN fortification may reduce anemia, iron deficiency anemia, and other micronutrient deficiencies slightly in infants, children, pregnant women [41]. Fortifying staple foods with vitamin A alone may have little to no difference to the risk of having subclinical vitamin A deficiency, but the evidence is very uncertain [46]. Compared with the provision of unfortified foods, the provision of staple foods fortified with vitamin A plus other micronutrients may decrease the risk of subclinical vitamin A deficiency, but the evidence is very uncertain. Similarly, there is very uncertain evidence that staple foods fortified with vitamin A plus other micronutrients may reduce the risk of subclinical vitamin A deficiency compared with no intervention [46].

### Malnutrition and infections

Moderate quality evidence indicated that LNS likely led to a clinically significant benefit in the number of children recovering from malnutrition compared with blended foods [47]. Kramer and Kakuma (2012) reported that exclusive breastfeeding (EBF) for six months led to a decreased risk of gastrointestinal infection, and there were no deficits in growth among infants; however, this was based on low quality evidence [17]. Low to moderate quality evidence suggested that children (between six months of age and five years) with moderate acute malnutrition were found to recover from moderate acute malnutrition when given specially formulated foods such as both LNS and blended foods when compared to standard care (medical care and counselling without food) [47]. Low to very low quality evidence indicated that vitamin D supplementation did not reduce the incidence of pneumonia and diarrhea among children under five years of age [48].

### Morbidity and mortality

Moderate quality evidence suggested that vitamin A supplementation (VAS) given to infants in the 1–6 months age group likely did not reduce mortality or morbidity [49]. In a review that included neonates at birth, high quality evidence suggested that VAS did not reduce mortality at 12 months of age. However, as per region-specific analyses, there was a significant decrease in the risk of death at six months among children in Asia, compared to no impact to a 21% rise in mortality risk in Africa [50]. For the outcome of diarrhea-related death, there was a high quality evidence that VAS significantly reduced (12%) mortality risk in the 6–59 month age group when compared to placebo or usual care [51]. Moderate quality evidence indicated that LNS likely did not decrease mortality or progression to severe acute malnutrition when compared with blended foods [47].

#### Community health services and health promotion

Community health services and health promotion interventions carried out in a wide range of contexts have shown some impact on a range of disease conditions.

### Mortality

Moderate quality evidence indicated that the administration of anthelminthic (or co-interventions) for soil-transmitted Helminth (STH) infections during the second or third trimester of pregnancy likely resulted in little to no difference on preterm births or perinatal deaths [52]. Moderate to high quality evidence indicated that neither single nor combined interventions reduced maternal deaths even as the latter strategy increased antenatal visits [53].

Low quality evidence showed that Integrated Management of Childhood Illness (IMCI) strategies including post-natal home visits may lead to lower neonatal and infant mortality [54]. Low quality evidence indicated that community-based delivery of antibiotics may slightly reduce neonatal mortality by treating neonatal Possible Severe Bacterial Infections (PSBI) as compared to standard care [55]. Community health educational interventions had a significant impact on decreasing overall neonatal mortality, early neonatal mortality, late neonatal mortality, and perinatal mortality; however, the quality of evidence varied from very low to low [56]. A systematic review examined community-based interventions (media campaigns, education, financial incentives for pregnant women to attend ANC care) and health systems interventions (including home visits for pregnant women by community health workers (CHWs). Low to moderate quality evidence indicated that single or combined interventions did not reduce the rates of perinatal or neonatal deaths [53].

### Child growth

High quality evidence showed that single community-based interventions and health systems interventions (including home visits for pregnant women by CHWs) did not reduce LBW [53]. Although, more women who got combined interventions had one or more antenatal visits, there were fewer LBW babies associated with combined interventions [53].

Low quality evidence suggested that nutritional education interventions given to pregnant women may slightly improve LBW compared to standard care or no intervention [39]. Another review reported that nutrition education given to pregnant women was found to slightly increase head circumference at birth. While birth weight among undernourished women improved, it did not significantly increase in the case of adequately nourished women [18]. Low to moderate quality evidence indicated that educational interventions improved complementary feeding and hygiene practices. However, it was reported that education improved the duration of EBF with community-based interventions but not with health facilities-based community-based interventions. The evidence was uncertain on the effect education on children’s’ growth [16]. Targeted client communication via mobile devices (TCCMD) may increase exclusive breastfeeding in settings where rates of exclusive breastfeeding are less common but have little or no effect in settings where almost all women breastfeed (low certainty in evidence). Low quality evidence showed that TCCMD may slightly increase EBF in settings where rates of EBF are less common but have little or no effect in settings where almost all women breastfeed [28].

### Vaccination status and uptake

Moderate quality evidence showed that IMCI strategies including post-natal home visits had little to no impact on measles vaccine coverage [54]. In another review, moderate quality evidence indicated that the use of LHWs was found to promote immunization uptake among children [57]. Low to moderate quality evidence suggested that face-to-face education may slightly improve the vaccination status of children and parents’ knowledge and their intention to vaccinate [58]. Limited and low quality evidence indicated that health education at home/village meetings probably led to an uptake of three doses of Diphtheria-Tetanus-Pertussis (DTP3) vaccines by more children [59]. There was evidence that providing information regarding the significance of vaccinations to parents during visits to the clinics and redesigned reminder cards on vaccination may enhance the uptake of three doses of the DTP3 vaccine [59].

### Anemia and Infectious diseases

Low quality evidence suggested that monthly administration of sulphadoxine-pyrimethamine (SP) was found to decrease maternal parasitemia and placental parasitemia at the time of delivery in HIV-positive pregnant women in their first or second pregnancy, living in malaria-endemic areas [60]. Low quality evidence showed that the administration of antihelminthics (or co-interventions) for STH infections during the second or third trimester of pregnancy on maternal anemia and pregnancy outcomes had little to no effect on maternal anemia in the third trimester [52].

Integration of HIV/AIDS and MNCHN-FP (Maternal, Neonatal and Child Health, Nutrition, and Family Planning) services likely had positive effects on contraceptive use, HIV testing, initiation of antiretroviral therapy in pregnancy; however, the quality of the evidence was low [61]. Low to moderate quality evidence indicated that educational programmes as single interventions probably had little to no effect on HIV, STI, and pregnancies among adolescents [62].

#### WASH (Water Sanitation and Hygiene) and Health Promotion

### Diarrhoea incidence and prevalence

Low to very low quality evidence indicated that education and hygiene promotion interventions with messages on disposal of child faeces might decrease diarrhea incidence by nearly 30% but did not affect diarrhea prevalence [37]. Evidence from interventions that addressed child faeces as part of a broader intervention directed at ending open defecation by all household members did not find an effect on diarrhea prevalence or STH infection [37]. Further, evidence showed that sanitation hardware (such as potties) and interventions relating to behavior change had mixed results on diarrhea prevalence, although no effect was seen [37]. Findings from the updated SR also reported that hand-washing promotion probably reduced diarrhea episodes by about 30% among communities living in LMICs [26]. Low quality evidence found that handwashing promotion may prevent almost 30% of diarrhea episodes in schools and about 28% of diarrhea episodes in communities in LMIC s [63].

### Child growth

Low quality evidence based on short-term studies indicated that WASH interventions (particularly provision of soap, solar disinfection of water, and improvement of water quality) showed a marginal benefit on linear growth of children aged under five years [64]. It was further reported that WASH interventions (specifically solar disinfection of water, provision of soap, and improvement of water quality) were shown to slightly improve height-for-age z-scores in children under five years of age [64]. Limited and low quality evidence showed no effect of slum upgrading on infant mortality but found that multicomponent slum upgrading led to a marginal reduction in the proportion of underweight children [34].

## Discussion

This overview examined interventions addressing MCH among urban poor populations, including homeless people in LMICs. It must first be mentioned that given the variation in definition and operationalization of populations, interventions and outcomes of interest, direct comparisons are likely to be misleading. We found no direct relevant evidence for pregnant and lactating homeless women (and children) in the reviewed literature. Most SRs that evaluated interventions relating to pregnant and lactating homeless women were conducted in HICs, which indicates a need to conduct more research in this domain in LMICs.

The results showed that there was mixed evidence of effect of slum upgrade, sanitation education and nutritional interventions on improving outcomes in urban poor pregnant and lactating women (and children) in LMICs [34, 36, 37, 39]. The range of nutritional interventions and outcomes varied across the SRs, making comparisons difficult. Mixed quality evidence showed that the range of nutritional interventions had little to no effect on child growth [35, 39–43]. Evidence indicated that MAM was treated effectively with LNS and blended foods [47]. However, evidence from Africa suggested that LNS compared to specially formulated fortified foods might be slightly more effective in aiding recovery from MAM and in weight gain among children aged 6 to 59 months [44]. There could be a host of reasons or confounders for this including the geographic context, the (base nutritional) content of blended foods and nutritional status of mothers during pregnancy. Supplementation combined with vitamins and minerals would help improve maternal and infant health rather than specific nutrients alone; however, outcomes varied by population, as a reduction in LBW rates was reported among adolescent pregnant women [65].

Supplementary feeding had little or negligible impact on the growth of children under five years of age [35, 42, 66]. However, supplementary feeding was found to be effective in terms of height and weight gain in children younger than two years old and more effective among poorer and less well-nourished children [66]. Evidence regarding the impact of MMN on anemia was mixed [40, 41, 45]. Other SRs found no significant benefit or differential impact of MMN compared to iron folate on third-trimester maternal anemia [67, 68]. Evidence indicated that child-feeding interventions were underperforming, with responsiveness to supplementary feeding being more among poorer and undernourished children. Supplementary feeding would be more effective if it is provided under supervision in a feeding centre, day care centre, or preschool. Children at day care centers or preschools had more benefit from the supplement [35].

There was no evidence of a reduced risk of mortality due to neonatal VAS on children less than one-year-old [49, 50, 68]. Similar findings were reported from another SR, with data from developed countries that showed that VAS had no effect in decreasing all-cause mortality in infants 1–6 months of age [69]. Based on data from developing countries, it was found that VAS reduced all-cause mortality (by 25 per cent) as well as diarrhea specific mortality (by 30 per cent) among children in the 6 to 59 months age group [70]. Another SR based on data from Asia, Africa and Latin America) showed that in children under 5 years old, VAS was associated with a decrease in diarrhea-related mortality (28 per cent) and a decrease in all-cause mortality (24 per cent) [71]. Further, the benefits of supplementation on mortality were seen to be greater in Asia compared to Africa and Latin America [71].

Vitamin D supplementation had no benefit on the incidence of pneumonia, [48] and in children under five with acute pneumonia [72]. Evidence from studies conducted in developing countries reported that factors such as accessibility (location, distance and transport), affordability (financial constraints) and cultural barriers constrained the uptake of ANC services [73]. Two SRs, one with evidence from Ethiopia [74] and another SR based on evidence from Sub Saharan Africa [75] found that urban residence, and women’s and husband’s education were associated with uptake of ANC services.

Three or more doses of sulphadoxine-pyrimethamine given to HIV positive women may have a marginal effect on the prevalence of maternal anemia and the number of LBW babies, [60] and IPT with three or more doses of SP was associated with higher birth weight and lower risk of LBW compared to standard 2-dose regimens among both HIV infected and uninfected women in sub-Saharan Africa [76]. Lindegren et al. (2012) reported a positive impact of integrating HIV/AIDS and MNCHN-FP services across settings [61]. This overview did not find any other literature on similar models of integrated services, but the included SRs showed that the integration of services may be feasible [77, 78]. Evidence from LMICs in Africa, the Caribbean, Europe, Asia that looked at strengthening linkages between FP and HIV interventions found that interventions that included a community component were feasible and effective [78].

There was mixed evidence on the effect of mass media campaigns and education on the uptake of ANC services [53]. Similar to the findings from Lewin et al.’s (2010) review [57], two other SRs, one based on studies from LMICs in Asia, Africa, and North America [79] and another based on findings from Brazil [80] found that CHW interventions were effective in improving breastfeeding [79, 80] as well as in reducing neonatal mortality in South Asian countries [81]. Continued uptake of ANC services depends on the positive experience of pregnant women with the health system, such as providing good quality, culturally sensitive services; however, barriers may include the indirect cost of services such as transport to the facility, cultural barriers relating to restrictions on movement, and lack of privacy [38].

The evidence on the impact of educational interventions on growth outcomes was mixed and generally of low quality, which indicated that educational interventions slightly improved immunization uptake and that redesigned reminder cards may enhance immunization uptake. In comparison, an SR suggested that educational interventions significantly increased childhood immunization uptake in LMICs, compared to HICs where the intervention was not consistently effective [82]. Evidence from both LMICs and developed countries indicated a positive impact of reminder strategies on immunization [82, 83]. Another SR found that breastfeeding education increased EBF rates, resulting primarily from community-based interventions, with those from LMICs showing a greater impact compared to HICs [84].

The effect of handwashing on diarrheal episodes differed based on location (school/community), specifically incidence. Evidence from less developed countries showed that handwashing reduced diarrhea illness [85], another SR conducted in HICs and LMICs found that handwashing promotion resulted in higher reduction of diarrhea than broader hygiene education [86]. Evidence from LMICs showed a significant association between WASH interventions and child growth [87] while another SR based on LMICs indicated that WASH interventions resulted in some reduction to no change in mortality [88].

The varying reportage of risk of bias for overlapping studies in different SRs was noted in this overview. It highlighted the need for greater rigour and consistency in the quality appraisal of reviews. The main limitation of our overview was the non-use of search terms related to individual LMIC countries. This may have resulted in the omission of some SRs. Further, given the relative dearth of literature, we are now persuaded to attempt a review rather than an overview for our population of interest, urban poor women and children as the literature is not as deep as we had anticipated when we began the overview. We had constraints of time in this exercise and moreover, were interested in the diversity of populations covered in existing reviews of MCH interventions (which we found to be quite poor!) [89].

There are some key policy implications of these findings. For one, the review suggests that even as MCH interventions have been the mainstay of public health interventions for decades; rather little is known about their impact on urban poor and homeless populations. Even as there remains work to be done with MCH interventions overall - in neglected areas like family planning, safe abortion care, and postpartum care of mothers- there is a need to generate primary evidence on what interventions work for urban poor and particularly homeless families.

## Conclusion

This review cast a wide net to try to see what interventions work for the MCH needs of the urban poor and homeless. Overall, the review did not find directly relevant information for the homeless. As regards the general urban poor population, there was a lack of emphasis on evidence related to family planning, safe abortion care, and postpartum care of mothers. There was mixed quality evidence that the range of nutritional interventions had little, unclear or no effect on several child mortality and development outcomes. From a policy perspective, this suggests that more research would be needed before promoting such interventions for urban homeless people. Interventions such as WASH, ensuring acceptability of community health services and health promotion type programs could be regarded as beneficial, although location seemed to matter. Overall, the generalizability of findings was poor, and there is a clear need for rigorous primary research on MCH interventions among the urban poor and homeless.

## Electronic supplementary material

Below is the link to the electronic supplementary material.

Supplementary Material 1

## Data Availability

All relevant data analysed during this review are included in this published article. Other relevant information extracted and analysed (such as data extraction tables) during the current review are available from the corresponding author on reasonable request.

## List of abbreviations

AMSTAR: A Measurement Tool to Assess Systematic Reviews
ANC: Antenatal Care
CHWs: Community Health Workers
DTP3: Diphtheria-Tetanus-Pertussis
EBF: Exclusive Breastfeeding
GRADE: Grading of Recommendations, Assessment, Development and Evaluations
HFA: Height for Age
HAZ: Height-for-Age Z scores
HICs: High-Income Countries
IFA: Iron-Folic Acid
IMCI: Integrated Management of Childhood Illness
JBI: Joanna Briggs Institute
LAZ: Length-for-Age Z scores
LNS: Lipid-based Nutrient Supplements
LMICs: Low- and Middle-Income Countries
LBW: Low Birth Weight
MCH: Maternal and Child Health
MNCHN-FP: Maternal, Neonatal And Child Health, Nutrition, and Family Planning
MMN: Multiple Micronutrient
PSBI: Possible Severe Bacterial Infection
PRISMA: Preferred Reporting Items for Systematic Reviews and Meta-Analyses
RCTs: Randomized Controlled Trials
SDG: Sustainable Development Goals
SHEWA-B: Sanitation Hygiene Education and Water Supply in Bangladesh
SRs: Systematic Reviews
STH: Soil-Transmitted Helminth
TCCMD: Targeted Client Communication via Mobile Devices
VAS: Vitamin A Supplementation
WASH: Water Sanitation and Hygiene
WAZ: Weight-for-Age Z scores
WHO: World Health Organization
